# Cloning, Expression and Biochemical Characterization of Endomannanases from *Thermobifida* Species Isolated from Different Niches

**DOI:** 10.1371/journal.pone.0155769

**Published:** 2016-05-25

**Authors:** Ákos Tóth, Terézia Barna, Erna Szabó, Rita Elek, Ágnes Hubert, István Nagy, István Nagy, Balázs Kriszt, András Táncsics, József Kukolya

**Affiliations:** 1 Department of Applied and Environmental Microbiology, National Agricultural Research and Innovation Centre, Budapest, Hungary; 2 Department of Genetics and Applied Microbiology, University of Debrecen, Hungary; 3 Department of Molecular Structural Biology, Max Planck Institute for Biochemistry, Martinsried, Germany; 4 Institute of Biochemistry, Biological Research Centre of the Hungarian Academy of Sciences, Szeged, Hungary; 5 Department of Environmental Protection and Environmental Safety, Szent István University, Gödöllő, Hungary; 6 Regional University Center of Excellence in Environmental Industry, Szent István University, Gödöllő, Hungary; Niels Bohr Institute, DENMARK

## Abstract

*Thermobifidas* are thermotolerant, compost inhabiting actinomycetes which have complex polysaccharide hydrolyzing enzyme systems. The best characterized enzymes of these hydrolases are cellulases from *T*. *fusca*, while other important enzymes especially hemicellulases are not deeply explored. To fill this gap we cloned and investigated endomannanases from those reference strains of the *Thermobifida* genus, which have published data on other hydrolases (*T*. *fusca* TM51, *T*. *alba* CECT3323, *T*. *cellulosilytica* TB100^T^ and *T*. *halotolerans* YIM90462^T^). Our phylogenetic analyses of 16S rDNA and endomannanase sequences revealed that *T*. *alba* CECT3323 is miss-classified; it belongs to the *T*. *fusca* species. The cloned and investigated endomannanases belong to the family of glycosyl hydrolases 5 (GH5), their size is around 50 kDa and they are modular enzymes. Their catalytic domains are extended by a C-terminal carbohydrate binding module (CBM) of type 2 with a 23–25 residues long interdomain linker region consisting of Pro, Thr and Glu/Asp rich repetitive tetrapeptide motifs. Their polypeptide chains exhibit high homology, interdomain sequence, which don’t show homology to each other, but all of them are built up from 3–6 times repeated tetrapeptide motifs) (PTDP-*Tc*, TEEP-*Tf*, DPGT-*Th*). All of the heterologously expressed Man5A enzymes exhibited activity only on mannan. The pH optima of Man5A enzymes from *T*. *halotolerans*, *T*. *cellulosilytica* and *T*. *fusca* are slightly different (7.0, 7.5 and 8.0, respectively) while their temperature optima span within the range of 70–75°C. The three endomannanases exhibited very similar kinetic performances on LBG-mannan substrate: 0.9–1.7mM of K_M_ and 80–120 1/sec of turnover number. We detected great variability in heat stability at 70°C, which was influenced by the presence of Ca^2+^. The investigated endomannanases might be important subjects for studying the structure/function relation behind the heat stability and for industrial applications to hemicellulose degradation.

## Introduction

The hemicellulose fraction of plant cell walls is mainly composed of xylan and mannan, and in case of leguminous plants high mannan content has also been found in seeds. Due to the complex structure of lignocellulose, hemicellulases are necessary for efficient extraction of cellulose from plant cell wall [1]. Biopolymers like cellulose or mannan are very promising raw materials for many industrial applications. Recently several studies have indicated that the most promising use of these biopolymers and their derivatives will be the health and food industry [2,3], since mannan degradation can produce a huge variety of biologically active oligosaccharides, which can be used as prebiotics.

Mannans are heterologous biopolymers with a very versatile composition. Galactomannan is composed of a homogenous backbone of β-1,4-linked mannose residues, that are branched with galactosyl residues, whereas galactoglucomannan has a heterogeneous backbone of β-1,4-linked glucose and mannose residues; in some cases (mainly in softwoods) this backbone is acetylated. The complete degradation of mannans requires a set of different enzymes. Endomannanases catalyze the random hydrolysis of the β -1,4-mannosidic backbone of the main mannan chain, α–galactosidases cleave the terminal α-1,6-linked D-galactosyl residues, and β-mannosidases hydrolyze β-1,4-linked mannose residues from the non-reducing ends of various oligosaccharides [4,5].

Some *Actinomyceta* like *Streptomyces* and *Cellulomonas* possess a large hydrolase pool, and several species can produce mannan degrading enzymes [6–9]. Endomannanases belong to three different glycoside hydrolase (GH) groups, namely the GH5, GH26 and GH76 according to the CAZY database [10] (http://www.cazy.org), and there are prokaryotic and eukaryotic endomannanases in each group. Glycoside hydrolases usually have modular architecture containing catalytic and carbohydrate binding modules (CBM), and their domain structures exhibit huge inter and intra species diversity [11]. Endomannanases may have different substrate specificity [12], and characteristic features of mannanases, such as the thermal stability are also affected by CBMs [13].

Enzymes with high thermal stability are of great interest for industrial applications [14]. One of the most promising sources of thermostable lignocellulolytic enzymes are compost inhabiting *Thermobifida* strains. These strains colonize the hot spots of the composts, and they are among the most effective organic matter degraders. *Thermobifida* genus belongs to the *Actinomycetales* order and four species, *T*. *fusca*, *T*. *alba* [15], *T*. *cellulosilytica* [16] and *T*. *halotolerans* have been described so far [17].

The genome of two *T*. *fusca* isolates, *T*. *fusca* YX [18] and *T*. *fusca* TM51 [19] have been sequenced. These genome sequences revealed 39 glycoside hydrolases belonging to different GH-families. Although the cellulolytic enzyme system in *T*. *fusca* is well characterized, our current knowledge about the hemicellulolytic enzyme system is fragmentary [20–22], and there are only a few studies about the other three *Thermobifida* species and their glycoside hydrolases. So far there are only three glycoside hydrolases—two endoglucanases and a xylanase—characterized from *T*. *halotolerans* [23–25]. A xylanase also has been described from *T*. *alba* [26], but there are no data available for glycoside hydrolases from *T*. *cellulosilytica*. Not only biochemical but also structural investigations of these enzymes are poor. The catalytic domain of *T*. *fusca* endomannanase has been investigated by Hilge et al. [27]. This study was the first publishing high resolution 3D structure of a mannan degrading enzyme, and assigned this endomannanase to the glycosyl hydrolase family 5 (GH5).

**Fig 1:**
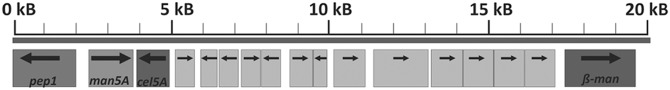
Genomic localization of the endomannanase gene of *Thermobifida fusca* strain TM51 according to the genome sequence data. Pale grey boxes without any marking represent conserved hypothetical protein encoding genes with unknown function and elements of peptide ABC transport system and transcriptional regulator genes, *pep1*: uncharacterized peptidase gene, *man5A*: endomannanase gene, *cel5A*: endoglucanase gene [30], *β-man*: beta-mannosidase gene [22]. On *T*. *fusca* genome the formerly published intracellular β-mannosidase is located in 13 kb proximity to the extracellular endomannanase.

Recently, two studies have been published focusing on the thermostability of the endomannanase from *T*. *fusca* and another endomannanase (StMan) from *Streptomyces thermolilacinus*. The first study showed that the thermal stability of these enzymes depends on the concentration of calcium ions [28], and the authors also determined the amino acid residues responsible for this phenomenon [29]. Formerly we had already described an intracellular mannobiose cleaving beta-mannosidase (ManB) from *T*. *fusca*, which seems to be the terminal part of the mannanase system [22]. Further exploring the genome sequence data, we identified two GH5 hydrolases—endomannanase (*man5A*) and endoglucanase (*cel5A*) genes—which are located on the genome upstream of the mannosidase (Fig 1) [30]. Here we report the characterization of the first endomannanase enzyme from *T*. *cellulosilytica*, and also a partial characterization of endomannanase from *T*. *halotolerans*. We also compared the three endomannanases from three different *Thermobifida* species namely from *T*. *fusca*, *T*. *cellulosilytica* and *T*. *halotolerans*.

## Materials and Methods

### Chemicals

Unless otherwise indicated, all chemicals herein used were analytical-grade and purchased from Sigma-Aldrich Ltd. (Budapest, Hungary).

### Microorganisms and culture conditions

Four *Thermobifida* strains were used in this study. *T*. *fusca* TM51 and *T*. *cellulosilytica* TB100 were isolated from the hot region of manure compost [31]. *T*. *halotolerans* YIM90462 and *T*. *alba* CECT3323 (synonym: *T*. *alba* ULJB1) were purchased from Japan Collection of Microorganisms (JCM) and Spanish Type Culture Collection (CECT), respectively.

### Cloning of endomannanases-encoding genes according to genome sequence data

Primers were designed using the genome sequence data of *T*. *fusca* TM51 [19]. Genomic DNA was isolated from each *Thermobifida* strains by MoBio UltraClean Microbial DNA Isolation Kit following manufacturer’s instructions. The endomannanase genes *man5ATf*, *man5ATc* and *man5ATh* were PCR-amplified with primers man5ATf forward (5’-GGTGCCATCATATGGCCACCGGGCTCC-3’), man5ATf reverse (5’- GTGCCATCTCGAGTCAGCGAGCGGTG-3’) and the four-fold degenerate primers man5ATfd forward (5’-GGTGCCATCATATGGCCACCGGGCTSS-3’) and man5ATfd reverse 5’-GTGCCATCTCGAGTCAGCGAGCGGWS-3’), with underlined sequences harboring *Nde*I and *Xho*I restriction sites. PCR reactions were carried out by using *Pfu* DNA polymerase (Thermo Fisher Scientific Inc.) for 32 cycles of 30 s at 94°C, 30 s at 60°C, and 3 min at 72°C, preceded by incubation for 5 min at 96°C. PCR-amplified fragments were digested with *Nde*I and *Xho*I enzymes (Thermo Fisher Scientific Inc.), ligated into the pET28a plasmid vector by using T4 DNA ligase (Thermo Fisher Scientific Inc.) and used to transform *E*. *coli* Top10 competent cells to isolate proper clones which were used for protein expression in *E*. *coli* BL21 (DE3) cells.

### Selection of endomannanase expressing *S*. *lividans* strains from an expression library

Genomic DNA from *T*. *halotolerans* YIM90462 *and T*. *cellulosilytica* TB100 was prepared as previously described [22] and partially digested with serial dilutions of *Sau*3AI (Thermo Fisher Scientific Inc.) for 1 hour at 37°C. Optimal enzyme concentration yielding the highest proportion of 10 kb DNA fragments was determined by agarose gel electrophoresis (0.6% agarose in TAE buffer) and DNA bands of this size were purified by Qiaquick gel extraction kit (Qiagen) according to manufacturer’s instructions. The fragments were then ligated into the *Streptomyces* vector pIJ699 digested with *BamH*I restriction endonuclease and treated with alkaline phosphatase to avoid self-ligation. Protoplast preparation, transformation, regeneration and selection of endomannanase-harbouring *Streptomyces* transformants were carried out as described previously [32]. Transformants were screened for endomannanase activity by growing the clones in 2 ml Luria-Bertani (LB) medium containing 200 μg/ml thiostrepton at 30°C, 200 rpm for 2 days, then culture supernatants were tested on agar plates containing 0.5% (w/v) LBG-mannan. Endomannanase activity was detected by Congo red staining after 30 min incubation at 50°C according to Posta et al. [33].

Plasmids from endomannanase-positive clones were purified and inserts were subcloned in *E*. *coli* TOP10 cells using pUC19 vector for sequencing purposes. Transformation and subsequent plasmid DNA purification were carried out as previously described [22]. Finally, full length endomannanase sequences were PCR-amplified and ligated into pET28 vector as described above by using the following primers: man5ATc forward, (5’-GGTGCCATCATATGGCGACCGGGATCCAC-3’), man5ATc reverse, (5’–GTGCCATCTCGAGTCAGTCGACGGAGCAGGTC-3’), man5ATh forward (5’–GGTGCCATCATATGGCCACCGGCTTC-3’) and man5ATh reverse (5’-GTGCCATCTCGAGTCAGTCGGTGGTG–3’).

### Phylogenetic analysis of *Thermobifida* strains and their endomannanases

For the molecular identification of investigated *Thermobifida* strains PCR amplification of 16S rDNAs were performed as described by Rainey et al. [34]. Partial sequences of the first 500 bp of the 16S rDNA were initiated with the 531r conservative eubacterial primer, almost-complete 16S rDNA sequences were determined by using primers 27f, 531r, 803f and 1492r [35]. 16S rDNA sequence reads and amino acid sequences of the cloned endomannanases were assembled in MEGA6 [36] then aligned by using the ClustalW algorithm. Neighbor-joining trees were constructed in MEGA5, performing 1000 bootstrap replicates.

### DNA sequencing and computer analysis

16SrDNA sequences and DNA fragments up- and downstream of endomannanases subcloned into pUC19 were determined with a DNA sequencer (ABI Prism 310, Perkin Elmer Co., USA).

DNA and protein endomannanase sequences were analyzed by using the BLAST server [37] and the MEGA6 software package [36]. Amino acid sequence and domain structure of ManB were determined by Swiss-Prot, EMBL and NCBI database queries and by using the Pfam [38] and InterPro [39] bioinformatics servers. Phylogenetic trees were reconstructed by the maximum likelihood method [40] by using the MEGA6 software package.

### Expression and purification of endomannanases

Recombinant His-tagged endomannanases were over-expressed in *E*.*coli* BL21 (DE3) cells. Transformants were grown at 37°C with 200 rpm aeration in 500 ml of LB medium containing 50 μg/ml kanamycin until optical density measured at 600 nm (OD_600_) reached 0.6–1.0. Protein expression was induced by adding isopropyl β-D-1-thiogalactopyranoside (IPTG, 1 mM final concentration), followed by overnight agitation at 20°C. Cells were harvested and disrupted by sonication and the lysate was centrifuged at 2,360 x *g* for 20 min at 4°C and supernatant was loaded on a 5 ml Hi Trap column (GE Healthcare) for immobilized metal ion affinity chromatography (IMAC) purification. Protein elution was performed with a 0–500 mM imidazole gradient in 300 mM NaCl, 20 mM sodium phosphate buffer, pH 7.2 and protein concentration of pooled fractions was determined by Bradford method using BSA as protein standard [41].

The molecular mass of the enzymes was estimated by SDS-PAGE analysis. Endomannanase zymography was done according to Posta et al. [33], with minor modification: instead of carboxymethyl-cellulose 0,1% LBG mannan was added to the gel.

### Biochemical characterization of endomannanases

Substrate specificity was assayed using different substrates, such as low viscosity carboxymethyl-cellulose (CMC), crystalline and micro-crystalline cellulose (MN300, Avicel), beech wood xylan and locust bean gum (LBG-mannan) as polysaccharides and 4-nitrophenyl β-D-mannopyranoside (pNP-βM) as an artificial aryl-mannoside substrate.

Endomannanase activities were determined on polysaccharide substrate by measuring liberated reducing sugars according Somogyi-Nelson method [42]. Dilution series of mannose stock solution were used for the determination of the reducing sugar calibration curves. Michaelis-Menten kinetic parameters of the endomannanases were estimated for LBG-mannan substrate in 10 different concentrations between 0–4 mg/ml (0, 0.2, 0.5, 1.0, 1.5, 2.0, 2.5, 3.0, 3.5, 4.0 mg/ml) at 50°C, in 50 mM sodium phosphate buffer, pH 7.5. The final volume of the enzyme reactions was 0.5 ml containing 1.4 μg/ml of Man5ATc, Man5ATh and of Man5ATf, respectively. Reactions were initiated by adding enzyme samples for the pre-incubated LBG-mannan substrate solutions at 50°C and then the incubation continued for further 5 minutes. All the measurements were carried out in triplicates. The initial rate of the enzyme catalysis was expressed in mM of reducing sugar liberated in one minute as it was determined from the calibration curve. The Michaelis-Menten kinetic constants were calculated by Origin 8.0 software program (OriginLab, Northampton, MA) using the Hill equation when the number of the occupied binding site was equal to one.

The pH dependence on endomannanase activity was measured in the presence of 2 mg/ml LBG substrate with different buffers in the pH range of 4–10 at 50°C, when the enzyme reactions contained 1.4 μg/ml of Man5ATc; Man5ATh and Man5ATf in 0.5ml reaction volumes. The following buffers were used (pH ranges are indicated in brackets): 100 mM citrate-phosphate (pH 4.0–6.5), 100mM sodium phosphate (pH 6.5–7.5), 200 mM Triethanolamine/HCl (pH 7.5–9.0) and 200 mM glycine/NaOH (pH 9.0–10.0).

The effect of temperature on endomannanase activity was determined in 50 mM sodium phosphate buffer, pH 7.5 at different temperature ranging from 40–90°C and using 2 mg/ml LBG-mannan substrate in 0.5 ml reaction volume. Enzyme concentrations were the same as in case of pH optimum determination. For both pH and temperature dependence studies the Somogyi-Nelson method was applied for the determination of the liberated reducing sugar concentration. Then, initial rates were calculated and converted into relative rates and plotted against pH and temperature.

Thermal stability of the three endomannanases was assessed at 70°C in either 100 mM Triethanolamine/HCl or 50 mM MOPS/NaOH buffer. For all the three enzymes, the effects of Ca^2+^ on thermal denaturation were investigated. In case of Man5ATh (50μg/ml), the effect of ion strength on thermal stability (NaCl at 0.2; 0.6; 1.0; 1.2; 1.6M concentrations) in MOPS/NaOH buffer (50mM, pH 7.5) with 5 mM Ca^2+^ was also determined at 70°C. The pH of the buffers was corrected by the temperature effect.

Man5ATh enzyme (50 μg/ml) in 50mM MOPS/NaOH buffer, pH 7.5, was incubated at 70°C in the presence and in the absence of Ca^2+^. MOPS/NaOH buffer (50 mM, pH 7.5) with added Ca^2+^ (5 mM) was also used when the salt effect on thermal denaturation of Man5ATh was studied in the presence of NaCl between 0–1.6M (0.2; 0.6; 1.0; 1.2; 1.6M) at 70°C.

Man5ATc enzyme (40 μg/ml) in 0.5 ml of 100 mM Triethanolamine/HCl buffer, pH 7.5, was incubated at 70°C in the presence and in the absence of Ca^2+^. In case of Man5ATf the same measurements in 100mM Triethanolamine/HCl buffer, pH 7.5 were performed as it was described for the Man5ATc enzyme. For all the three enzymes the rate of thermal unfolding was also followed when Ca^2+^ was replaced by 2mM EGTA at 70°C.

In all cases, from the incubated endomannanase solutions, time course aliquots (10 μl) were withdrawn, cooled on ice for at least 30 min and then assayed for endomannanase activity at 50°C in the presence of 2 mg/ml LGB-mannan in 50 mM phosphate buffer by Somogyi-Nelson method. The residual activity was calculated as a fraction of the initial activity and plotted against time. The data were fitted to one-step transition mechanism between two states, the native and the denatured ones (E→E_D_). The single step is assumed irreversible and to follow first order kinetics. In this mechanism, the thermal inactivation rate constant (min^-1^) was assessed from a single exponential decay curve, d[E]/t = -k_D_[E]. The active enzyme concentration can be expressed as the enzyme activity, A_t_, after heat treatment for a given period of time and the initial activity, A_0_. The integration of the equation then gives lnA_t_/A_0_ = k_D_, where A_t_/A_0_ is the residual activity. ORIGIN software was used for the data analysis and graphic representation. Half-life t_1/2_ is derived by the following equation t_1/2_ = ln2/k_D_.

### Nucleotide sequence accession numbers

GenBank accession numbers of determined endomannanase nucleotide sequences are as follows: *man5A*Tf: KF684964, *man5A*Tc: KF684965, *man5A*Th: KF684966, *man5A*Ta: KP889060. 16S rDNA sequence accession numbers are: *T*. *alba* CECT3323: KP410834, *T*. *alba* JCM3077: AF002260, *T*. *cellulosilytica* TB100: NR_025438, *T*. *halotolerans* YIM90462: NR_044446, *T*. *fusca* ATCC27730: AF028245, *T*. *fusca* TM51: AOSG01000000.

GenBank accession numbers of the three identified endomannanase amino acid sequences are: Man5ATf: AHB89702, Man5ATc AHB89703 and Man5ATh AHB89704.

## Results and Discussion

### Endomannanase cloning based on genome sequences

PCR production of endomannanase genes from four thermobifida strains were probed by homologous and degenerated primers. The primer design was based on the complete genome sequence of *T*. *fusca* TM51. 1362 bp DNA fragments were synthesized when *T*. *fusca* and *T*. *alba* genomic DNA served as template, but in case of *T*. *halotolerans* and *T*. *cellulosilytica* no PCR products were obtained in spite of extensive PCR optimization experiments. The obtained PCR products were named *man5A*Tf and *man5A*Ta and since they showed 99% DNA and 100% aminoacid (AA) homology investigations were limited to *man5A*Tf which was cloned into the pET28a expression vector.

### Cloning endomannanases from expression libraries

To capture endomannanase genes of *T*. *halotolerans* YIM90462 and *T*. *cellulosilytica* TB100, expression libraries were generated in *Streptomyces lividans* TK24 strain. Genomic DNAs were partially digested with *Sau*3AI endonuclease and DNA fragments of 10 kb were cloned into the pIJ699 vector. After transformation, thiostrepton resistant clones were selected. Clones of each library were screened on LBG containing agar plates. Endomannanase over-producing colonies were detected by Congo red staining method.

Plasmids were isolated from the mannanase positive clones and the inserts were fully sequenced after subcloning. Sequence analyses revealed a 1368 bp *man5A*Th gene from *T*. *halotolerans* and a 1320 bp *man5A*Tc gene from *T*. *cellulosilytica*. Specific primers were designed for the identified endomannanase genes and used in PCR reactions to obtain DNA fragments having *Nde*I and *Xho*I cloning sites for cloning into the corresponding sites of pET28a vector. By this method 6His tag for affinity purification was introduced on the N-terminus of both genes.

### Phylogenetic analysis of thermobifida endomannanases

**Fig 2:**
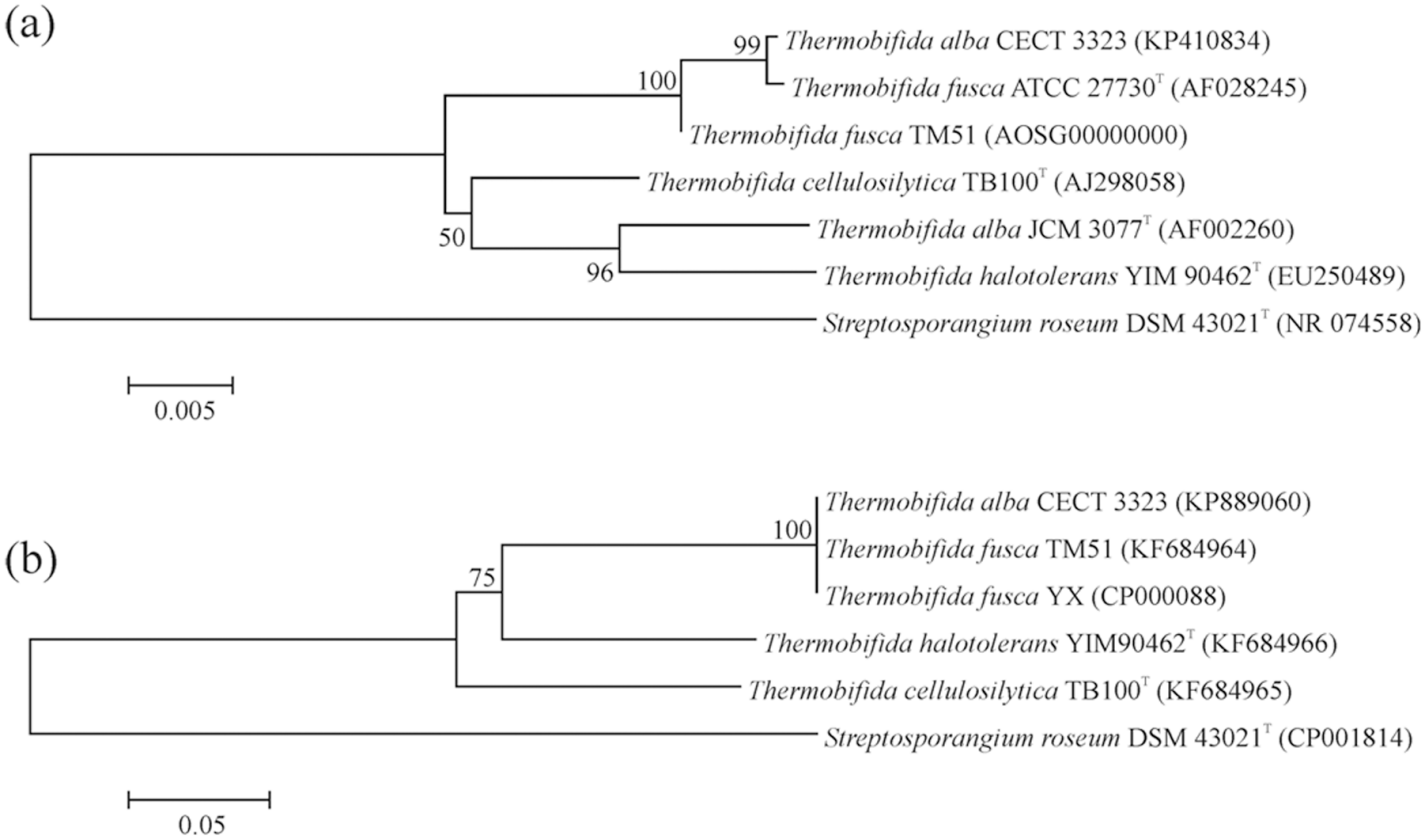
Phylogenetic analysis of investigated thermobifida strains and their endomannanase enzymes. The upper tree (a) represents the phylogenetic relation of *T*. *alba* CECT3323 and *T*. *fusca* TM51 compared to other thermobifida type strains (*T*. *fusca*, *T*. *cellulosilytica*, *T*. *halotolerans* and *T*. *alba*) of the genus using 16S rDNA sequences. The lower tree (b) represents phylogenetic relations of Man5A enzymes of *T*. *fusca* TM51, *T*. *halotolerans* YIM90462, *T*. *alba* CECT3323 and *T*. *cellulosilytica* TB100 based on their AA sequence. *Streptosporangium roseum* DSM 43021^T^ acts as an outgroup strain in both trees. Both phylogenetic trees indicate that *T*. *alba* CECT3323 is a *T*. *fusca* species.

The four investigated strains were taxonomically characterized by 16S rDNA sequence analysis. The obtained phylogenetic tree indicates that *T*. *alba* CECT3323 clashes to one cluster with type strain *T*. *fusca* ATCC27730^T^ and *T*. *fusca* TM51, and is clearly separated from type strain *T*. *alba* (Fig 2). When the endoxylanase of *T*. *alba* CECT3323 was published [26] the strain was characterized by phenotypic characteristics and so far there are data on hydrolases only from this strain. The nucleotide sequence alignment between XylA of *T*. *alba* CECT3323 (Z81013) and *T*. *fusca* YX xylanase (AAZ56956) revealed 100% homology in the catalytic and carbohydrate binding module [26,43]. Accordingly, the Man5ATa enzyme of *T*. *alba* CECT3323 shows 100% identity to the Man5ATf enzyme of *T*. *fusca* TM51 (Fig 2). Based on enzyme identities and molecular taxonomy results we concluded that the CECT3323 strain belongs to *T*. *fusca* species therefore the Man5ATa enzyme was excluded from further biochemical analysis.

**Fig 3:**
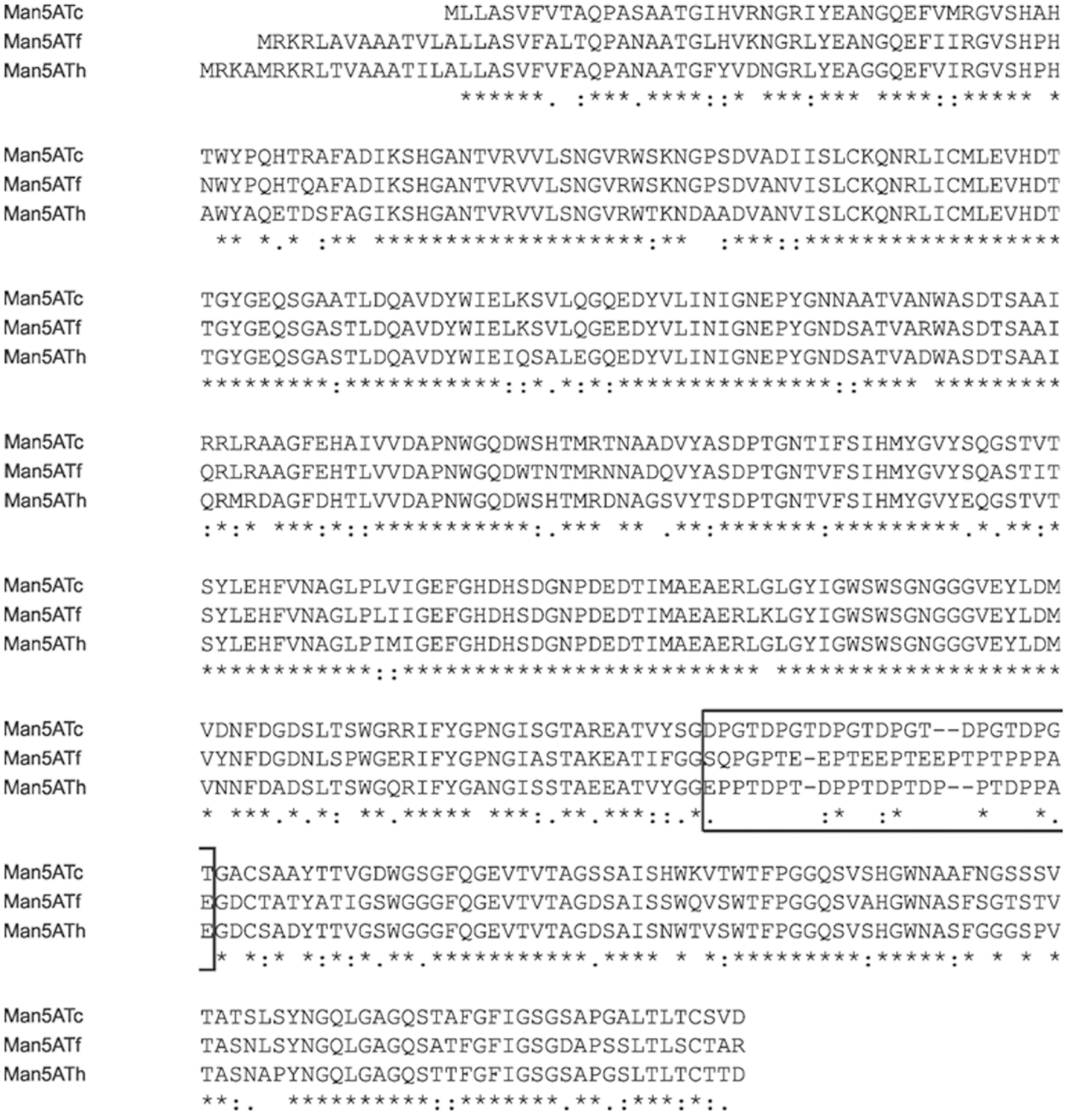
Representation of AA similarity and domain orientation of investigated endomannanases. AA sequences of Man5Atc, Man5ATf and Man5ATh endomannanases from *T*. *cellulosilytica*, *T*. *fusca* and *T*. *halotolerans*, respectively, are aligned. N-terminal GH5 catalytic and C-terminal polysaccharide binding modules (CBM2) are separated by the linker sequences (boxed region). Symbols: * - indicates positions which have a single, fully conserved residue, : - indicates conservation between groups of strongly similar properties, . - indicates conservation between groups of weakly similar properties.

The lengths of the *man5ATf*, *man5ATc* and *man5ATh genes* are 1362, 1320 and 1368 bp, respectively. We determined the signal peptides for secretion by SignalP software (http://www.cbs.dtu.dk/services/SignalP/), and consequently we cloned the endomannanases containing 425 (Man5ATf), 424 (Man5ATc) and 423 (Man5ATh) aminoacids, without signal peptides. The phylogenetic analysis of AA sequences revealed that endomannanases of *T*. *halotolerans* and *T*. *cellulosilytica* are much closer to each other than to *T*. *fusca* (Fig 2). There is high similarity among amino acid sequences of mature enzymes (93–96%); the identity between Man5ATf and Man5ATc, Man5ATf and Man5ATh, and Man5ATc and Man5ATh is 79, 81 and 82%, respectively (Fig 3). Mature proteins consist of N-terminal GH5 catalytic domain and C-terminal carbohydrate binding module (CBM2). The identity between catalytic domain pairs is between 84–86%, while we found lower homology values for the CBM domains 74–83%, although the similarity is still very high (93–97%). The highly variable inter-domain 23–25 AA linker sequences show a variation of prolin, threonine and aspartate/glutamate rich 3–6 times repetitive tetrapeptide motives: 5xPTDP-*Tc*, 3xTEEP-*Tf* and 6xDPGT-*Th* (Fig 3).

Similar linker regions also can be found in cellulases of *T*. *fusca* YX and their role has been investigated by posttranslational modification [20]. A protease was identified cleaving the Cel9A intact enzyme along the linker sequence producing catalytic and CBM domains [44]. The substrate specificity of the enzyme without the CBM domain was changed; its activity increased towards shorter oligosaccharide fractions. The genome of thermobifida strains is relatively small, and instead of producing a larger enzyme set this posttranslational mechanism makes their polysaccharide degrading capability more diverse and effective. The different linker sequences in homologous enzymes of thermobifida species (that populate same niches) most probably provides a control over posttranslational modifications to make thermobifidas more competitive in the race for available substrates.

### Heterologous expression and purification of endomannanases

**Fig 4:**
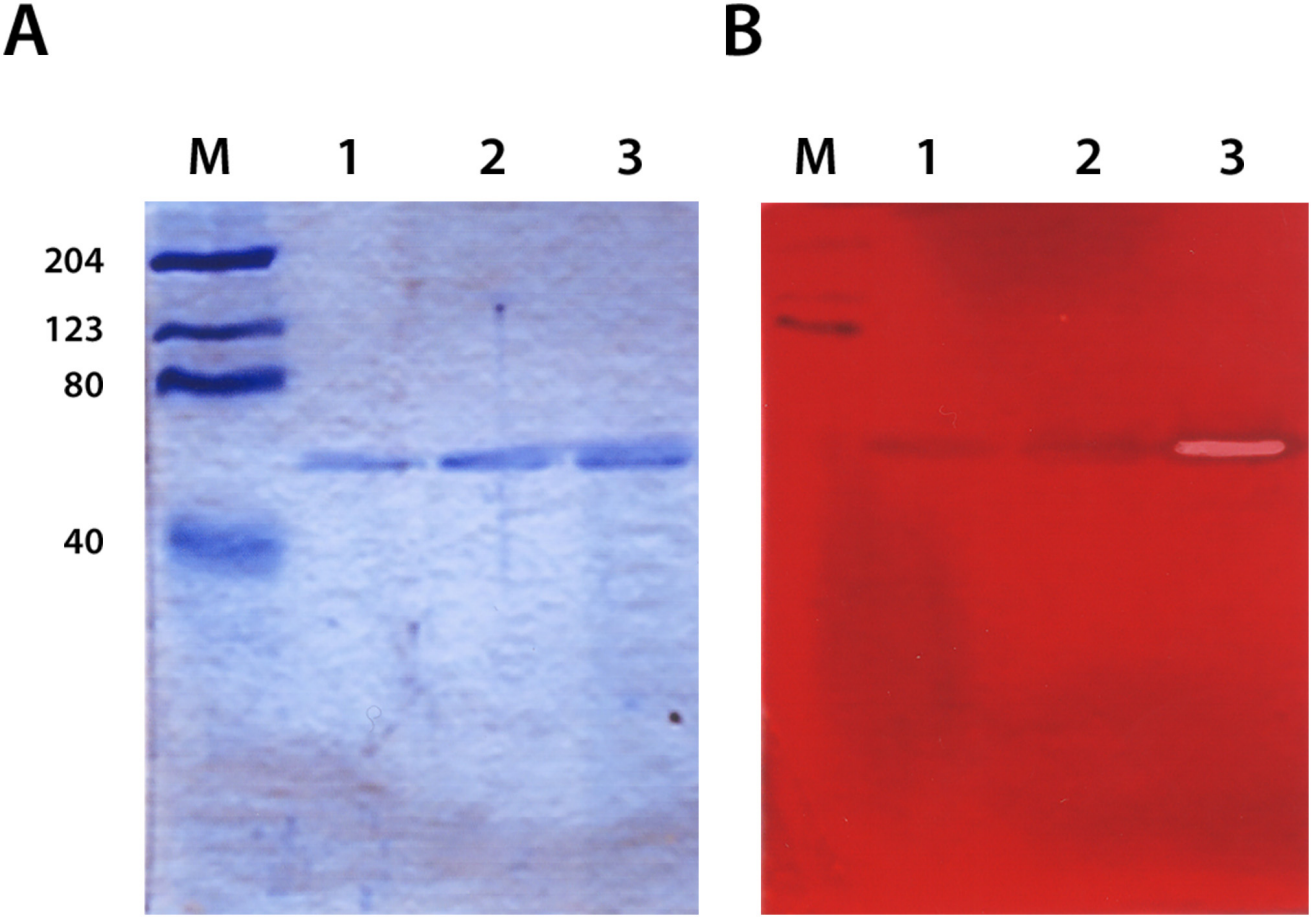
SDS-PAGE separation and activity staining of expressed endomannanases. Left panel: Coomassie-blue stained SDS-PAGE; right panel: endomannanase zymogram. M: molecular weight marker; 1: Man5ATh; 2: Man5ATc; 3: Man5ATf. The molecular weight of the three endomannanases almost identical, in the size range 48–50 kDa. Zymogram shows activity in the case of the highly stable endomannanase of *T*. *fusca*.

Man5ATc, Man5ATf and Man5ATh endomannanases were expressed in *E*. *coli* BL21 (DE) and the yield from 1 L culture after IMAC affinity chromatography purification was as follows: Man5ATh 25 mg, Man5ATc 33 mg and Man5ATf 45 mg. SDS PAGE analysis of expressed proteins indicated 48–50 kDa size that was in good agreement with theoretical molecular weights (Fig 4). Isoelectric points were predicted using the algorithm of Kozlowski (2013, http://isoelectric.ovh.org/). The most acidic protein is Man5ATh (pI 4.102) while Man5ATf and Man5ATc are less acidic with almost identical values (pI 4.519 and 4.567, respectively).

### Biochemical characterization of Man5ATc, Man5ATf and Man5ATh β-1,4-endomannanases

#### Substrate specificity measurements

The GH5 endomannanase family includes several types of hydrolases, among others endocellulases, glucosidases, xylanases and mannanases. Substrate specificities of expressed endomannanases were tested with carboxymethyl-cellulose (CMC), crystalline and micro-crystalline cellulose (MN300, Avicel), beech wood xylan, pNp-mannopiranozid and locust bean gum (LBG). All the investigated Man5A were active only on LBG. This narrow substrate specificity indicates that the axial OH group at C2 on the pyranose ring is essential in ligand binding at the active site and suggests a potential biotechnological application of the enzymes in the production of oligomannan prebiotics [5].

#### Kinetic studies

Endomannanase activities of the three GH5 glycoside hydrolases were investigated on LBG-mannan substrate. The applied substrate consists of a β-(l,4)-linked mannan backbone with single α-(l,6)-linked galactose side chains. The endomannanase activity of the three GH5 glycoside hydrolases were not affected by the presence of chelating agents such as EDTA or EGTA suggesting that the catalytic effect of the enzymes did not depend on metal ions.

**Fig 5:**
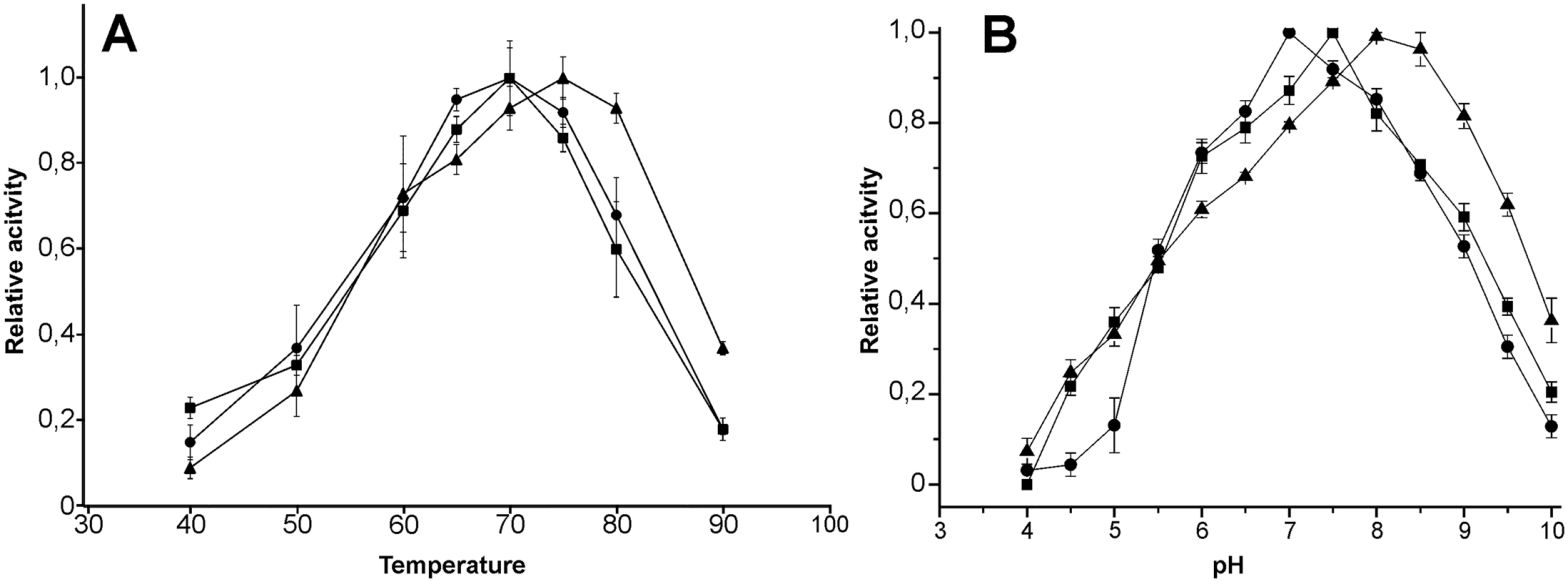
The effect of temperature and pH on enzyme activity of *Thermobifida* endomannanases. The pH and thermal optimum determination studies were done in the presence of 2 mg/ml LBG-mannan. Symbols: ●-Man5ATc, ■-Man5ATh, ▲-Man5ATf. A: The temperature optima of the endomannanases are increasing in the following order Man5ATh, Man5ATc and Man5ATf with values of 70°C and 75°C, respectively. B: The functional pH range of Man5ATc and Man5ATh endomannanases overlaps in the range of 5.5–9.0 and slightly differs from Man5ATf. The pH optima are 7.0 (Man5ATc), 7.5 (Man5ATh) and 8.0 (Man5ATf), respectively.

The temperature optima of the *Thermobifida* endomannanases are in the range of 70–75°C (70°C for Man5ATc and Man5ATh; 75°C for Man5ATf) at given assay conditions (Fig 5) and this value classifies them to high temperature optimum enzymes. Endomannanases from the eubacterial *Caldibacillus cellulovorans* [45] and the archaeon *Thermotoga neapolitana* [46] have significantly higher temperature optimum: 85°C. The CBM domain free catalytic domain of endomannanase of *T*. *fusca* KW3 was also characterized as thermophilic enzyme (80°C) but it can’t be compared to our values as a significant portion of the enzyme was deleted [27]. The recently described endomannanase from *T*. *fusca* BCRC19214 which was expressed in *Yarrowia lipolytica* has a very similar thermal optimum at the 75–80°C range [47].

The functional pH range of Man5ATc and Man5ATh endomannanases completely overlaps in the range of 5.5–9.0 (where more than 50% of maximum activity was detected) with pH optimum of 7.0 and 7.5 for Man5ATc and Man5ATh, respectively. Man5ATf possesses the wildest working pH range of 5.5–9.7 with pH optimum of 8.0 (Fig 5). With these slightly basic values thermobifida Man5A enzymes form a distinct sub-group in the microbial GH5 endomannanase family. Endomannanases of bacilli have more basic values around pH 9 [48–50] and most of the prokaryotic endomannanase enzymes from *Streptomyces lividans*, *Clostridium cellulovorans*, *Vibrio* sp. and *Geobacillus stearothermophilus* have activity maximum at neutral pH [6,51–53]. Fungal GH5 endomannanases are adapted to acidic environment [54–56]. Michaelis-Menten kinetic parameters determined at pH 7.5 and 50°C are listed in Table 1. Kinetic performances expressed in catalytic constants (k_cat_) are similar for all the three investigated mannanases with the value of 100±20 sec^-1^. Slight difference found in the individual Michaelis-Menten constants, Man5ATc has the highest affinity toward the carob-mannan substrate with K_M_ value of 0.84 mg/ml (Table 1). Endomannanases from *Aspergillus niger BK01* and from *Bacillus sp*. *MG-33* have higher affinity toward locust bean galactomannan with Km value of 0.6 mg/ml and 0.16 mg/ml, respectively [57,58]. The majority of the endomannanases from either fungi or bacilli taxa exhibited considerable lower affinity for this type of mannan like endomannanases from *Bacillus licheniformis* and from *Penicillium oxalicum GZ-2* with Km values of 14.9 mg/ml and 7.6 mg/ml, respectively [59,60].

**Table 1:** Michaelis-Menten kinetic parameters of endomannanases for LBG-mannan substrate.

Endomannanase	K_M_ (mg/ml)	k_cat_ (s^-1^)	k_cat_/K_M_ (ml s^-1^ mg^-1^)
**Man5ATf**	**1.65**±**0.40**	**122±11**	**74**
**Man5ATh**	**1.30 ±0.30**	**78±9**	**60**
**Man5ATc**	**0.84±0.15**	**89±5**	**106**

Kinetic studies were performed in 50 mM phosphate buffer, pH 7.5, using LBG-mannan as substrate in the concentration range of 0–4 mg/ml at 50°C. Man5ATc has the highest affinity toward the carob-mannan substrate with K_M_ value of 0.84 mg/ml.

#### Thermostability studies

Denaturation kinetics of the three mannanases were studied at 70°C in the presence and in the absence of Ca^2+^ in order to investigate the thermostability of the enzymes and the influence of this metal ion on thermal unfolding. For all the three endomannanases the unfolding kinetics obeys a single step exponential decay. The heat inactivation constants (k_D_) and the calculated half-lifes (t_1/2_) determined in the different conditions were summarized in Table 2. The unfolding kinetics of *T*. *fusca* and *T*. *halotolerans* enzymes were affected by Ca^2+^ as their thermal stability has increased significantly: doubled their life-times when the enzyme solutions contained 5mM of metal ion. The stabilizing effect of Ca^2+^ ion against thermal denaturation has also been detected in the case of the catalytic domain of *T*. *fusca* endomannanase by Kumagai et al. at different temperatures with comparable results [28]. The key residues in calcium binding have been identified in case of *T*. *fusca* mannanase [29] and the same motif (Asp-264, Glu-265, and Asp-266) was present also in the other investigated mannanases (Man5ATc and Man5ATh). Interestingly, the thermal denaturation of Man5ATc was not influenced by Ca^2+^ despite the existence of the putative Ca^2+^ binding motive (Table 2). The effect of salt on thermal denaturation of Man5ATh was investigated in the presence of NaCl in the concentration range of 0–1.6M. The unfolding kinetics were not affected significantly by the presence of NaCl when its concentration was not higher than 0.8M. However, when the NaCl concentration was increased to 1.6 M, the speed of unfolding tripled compared to the level when there was no salt in the system (Table 3). These results suggest that Man5ATh is a moderately halophilic protein, which is in a good agreement with the original habitat (a salt mine) of *T*. *halotolerans* YIM90462 strain [17].

**Table 2:** Heat inactivation kinetic constants and the derived half-life for the thermal unfolding process of endomannanases at 70°C.

	k_D_ (min^-1^) in the presence of Ca^2+^	t_1/2_ (min) in the presence of Ca^2+^	k_D_ (min^-1^) in the absence of Ca^2+^	t_1/2_ (min) in the absense of Ca^2+^
Man5ATf	5.6 *10^**-3**^ ±4*10^**-4**^	**123**	1.29 *10^**-2**^ ±3.9*10^**-4**^	**54**
Man5ATc	3.3 *10^**-2**^ ±2.5*10^**-3**^	**21**	3.5 *10^**-2**^ ±1.7*10^**-3**^	**20**
Man5ATh	1.9 *10^**-2**^ ±1.8*10^**-3**^	**36**	4.0 *10^**-2**^ ±4.5*10^**-3**^	**17**

Thermal unfolding was protected by the presence of Ca^2+^ in the case of Man5ATf and Man5ATc.

**Table 3:** Salt concentration effect on the heat inactivation constant for the thermal denaturation of *T*. *halotolerans* endomannanase at 70°C.

NaCl (M)	k_D_ (min^-1^)	t_1/2_ (min)
0.4	2.4*10^**-2**^ **min**^**-1**^ ±2*10^**-3**^	**29**
0.8	2.5*10^**-2**^ **min**^**-1**^ ±2*10^**-3**^	**28**
1.2	3.8*10^**-2**^ **min**^**-1**^ ±2*10^**-3**^	**18**
1.6	5.2*10^**-2**^ **min**^**-1**^ ±5*10^**-3**^	**13**

In thermal unfolding Man5ATh is able to tolerate the presence of NaCl as far as the level of 0.8M.

**Fig 6:**
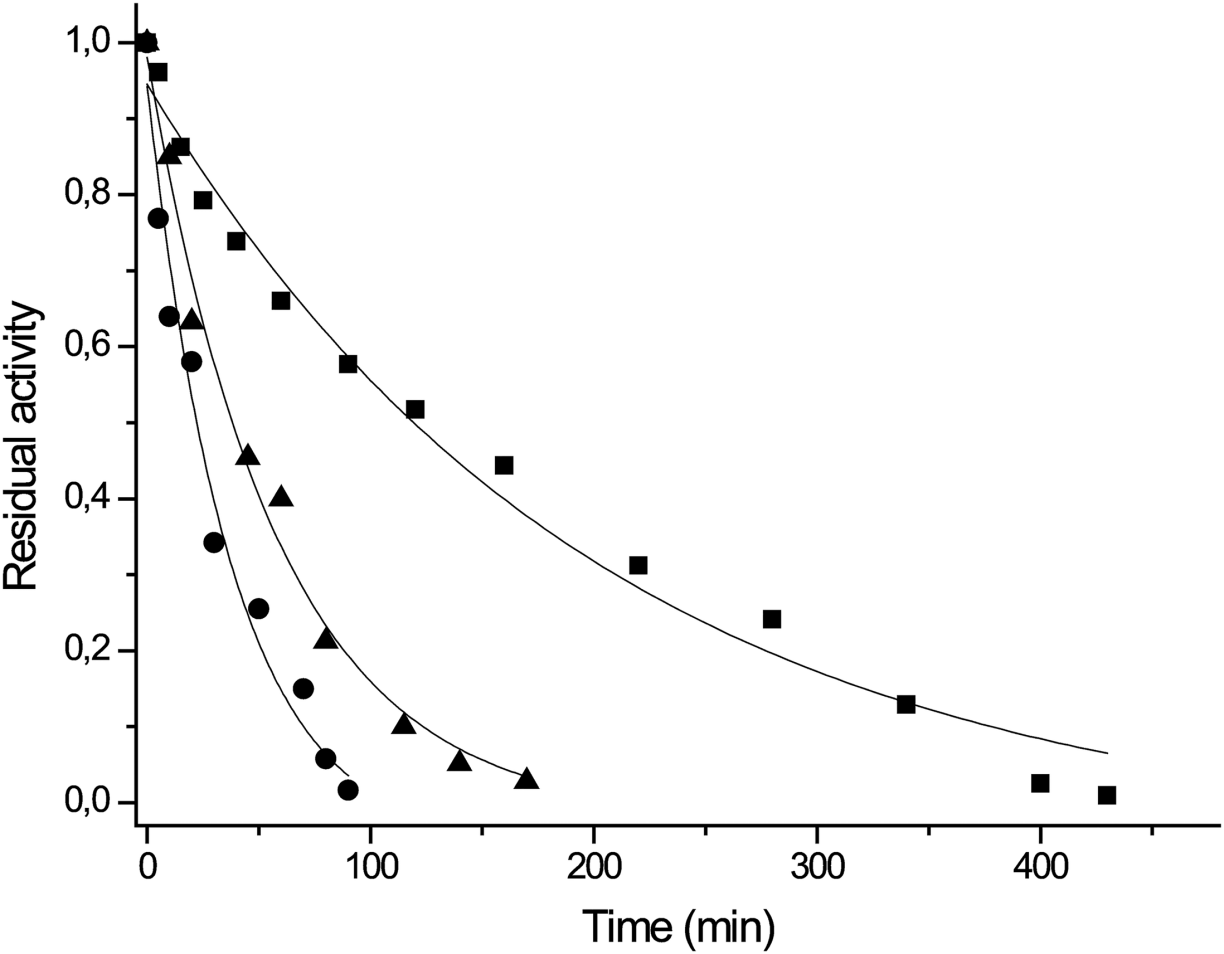
Thermostability of thermobifida endomannanases at 70°C measured at optimal conditions. Symbols: ■-Man5ATf in 50 mM Trietanolamin/HCl pH7.5 in the presence of 5 mM Ca^2+^; ▲-Man5ATh in 50 mM MOPS pH 7.5 in the presence of 5mM Ca^2+^ and 0,4 mM NaCl; ●-Man5ATc in Trietanolamin/HCl pH7.5 in the presence of 5mM Ca^2+^. The half life of Man5ATf, Man5ATh and Man5ATc were 123 min, 36 min and 21 min, respectively.

In our measurements, *T*. *fusca* β-(l,4)-endomannanase was proved to possess the greatest thermostability among the investigated enzymes with a life-time of 123 min^-1^ at 70°C in the presence of Ca^2+^ (Fig 6).

Alterations in mannanase stabilities can be explained with differences in environmental factors of niches populated by investigated thermobifida strains. *T*. *fusca* TM51 was isolated from moderately basic (pH 8.5) compost [31] and its temperature optimum is 60°C. The other two strains, *T*. *halotolerans* and *T*. *cellulosilytica* have considerably lower temperature optimum (50–55°C). The half-life value of Man5ATf for 70°C (123 min) places the enzyme into an elite group of highly stable mannanases together with robust enzymes from the archaeon *Thermotoga thermarum* (half life 120 min at 90°C) and the eubacterium *Caldibacillus cellulovorans* (half life 48 min at 85°C, and no loss in activity after 24 h at 70°C) [45,61].

## Conclusion

Endomannanase genes from thermobifida strains were isolated whose recombinantly expressed polysaccharide degrading enzymes have been partially characterized. The molecular taxonomy investigations carried out clearly indicated that *T*. *alba* CECT3323 strain was previously miss-classified and in the reality it is a *T*. *fusca* strain, therefore at present time there are no data on *T*. *alba* hydrolases.

Investigated endomannanases from *T*. *fusca*, *T*. *cellulosilytica* and *T*. *halotolerans* belong to GH5 hydrolases. They have modular architecture and have polysaccharide binding site at the C-terminus. The AA homology between them is 82–84% and their characteristics are very similar regarding the kinetic parameters although the pH and temperature optima are slightly different. The differences in thermal stability of the three enzymes are more pronounced: the life-time of Man5ATf is four-five times higher at 70°C compared to the other two enzymes. Man5ATh of *T*. *halotolerans* moderately salt tolerant, its thermal stability is preserved up to 0.8M of NaCl concentration. These parameters coincided well with environmental parameters of niches where these thermobifida strains were isolated from.

Despite the high sequence similarity of the investigated mannanases they exhibit different temperature stability, and this can be a starting point for further structural-functional investigations and for industrial applications to produce biologically active, oligomannan prebiotics.
